# Interpreting imported malaria genomes: population-genomic sensitivity analyses of West Africa-associated *Plasmodium falciparum* isolates detected in China

**DOI:** 10.3389/fcimb.2026.1950822

**Published:** 2026-10-12

**Authors:** Xuli Du, Ye Hu, Haimo Shen, Mengyu Li, Yuxin Wang, Yanli Bai, Shenbo Chen, Yanting Fan

**Affiliations:** 1 Department of Parasitology, School of Medicine, and Department of Blood Transfusion, Xi’an International Medical Center Hospital, Northwest University, Xi’an, Shaanxi, China; 2 National Key Laboratory of Intelligent Tracking and Forecasting for Infectious Diseases, National Institute of Parasitic Diseases, Chinese Center for Disease Control and Prevention (Chinese Center for Tropical Diseases Research), National Health Commission of the People’s Republic of China (NHC) Key Laboratory of Parasite and Vector Biology, World Health Organization (WHO) Collaborating Center for Tropical Diseases, National Center for International Research on Tropical Diseases, Shanghai, China; 3 College of Life Sciences, Northwest University, Xi’an, Shaanxi, China

**Keywords:** genomic surveillance, imported malaria, malaria elimination, *Plasmodium falciparum*, sampling bias

## Abstract

**Background:**

Genomes obtained through imported-malaria surveillance can support source attribution and monitoring of parasite movement. However, these genomes are sampled after travel, diagnosis, reporting, and specimen availability, and may not be directly interchangeable with parasites sampled in endemic settings. Comparisons with public reference data may also be affected by geographic composition, sequencing quality, genotype filtering, and within-host infection complexity.

**Methods:**

We compared West Africa-associated *P. falciparum* isolates detected through China’s post-elimination malaria surveillance system with West African *Pf3K* and locally collected comparison isolates. We implemented country-balanced reference subsampling, a geographically restricted and quality-controlled population-structure analysis, and sensitivity analyses restricted to imported isolates with F_WS_>0.90 or 0.95. Population structure, within-host complexity, identity-by-descent, nucleotide diversity, gene-level F_ST_, and Tajima’s D were evaluated using analysis-specific variant sets.

**Results:**

In the quality-controlled D1 analysis, 31 China-imported isolates showed a population-structure distribution that did not fully overlap with the selected West African *Pf3K* reference panel. This broad placement was qualitatively compatible with the high-F_WS_ D2a subset of four imported isolates. Country-balanced reference subsampling produced similar broad PCA placement. In the D2b analysis of nine imported isolates with F_WS_>0.90, central gene-level F_ST_ summaries were like those in the original analysis, whereas individual-gene estimates and Tajima’s D contrasts were sensitive to the revised filtering and sample restriction.

**Conclusions:**

Imported malaria genomes provide a surveillance-defined view of parasite diversity and remain useful for regional population placement and surveillance applications. However, their comparison with endemic reference panels is sensitive to dataset composition, variant filtering, and infection complexity. Imported surveillance genomes should therefore not be interpreted as unbiased or directly interchangeable proxies for parasite populations in presumed source settings. The observed differences cannot be attributed solely to surveillance pathway.

## 1 Introduction

Genomic surveillance is increasingly recognized as a core component of modern infectious-disease surveillance (Neafsey et al., 2021; Akimkin et al., 2024). The WHO frames pathogen genomics as a public-health capacity for detecting, monitoring and responding to infectious threats, rather than as a purely research-oriented activity (World Health Organization, 2022). In malaria, this broader pathogen-surveillance agenda has been translated into parasite- and vector-specific questions (Neafsey and Volkman, 2017). The WHO explicitly considered how genetic data can be used by malaria programs, while malaria genomics reviews have argued that parasite genomic data can support elimination by tracking parasite movement, transmission connectivity, drug resistance, diagnostic escape, vaccine-relevant variation and population change (Committee, 2019). Recent implementation-focused studies further show that malaria genomic surveillance is becoming operationally feasible, including targeted nanopore sequencing approaches designed to generate policy-relevant information on antimalarial resistance, diagnostic failure markers, complexity of infection and vaccine targets from field-compatible samples (Nsanzabana, 2019).

This surveillance rationale is particularly relevant after malaria elimination. In post-elimination settings, the central public-health task is to sustain malaria-free status by detecting imported infections, preventing onward transmission and rapidly investigating epidemiologically unusual cases (Golumbeanu et al., 2025). China was certified malaria-free by WHO in 2021 after reporting zero indigenous cases for consecutive years, but WHO’s certification process also emphasizes the need to prevent re-establishment after elimination (Cao et al., 2021). In this context, imported malaria becomes a major surveillance category. Imported malaria has been defined as infection acquired in a malaria-endemic area with clinical manifestation, diagnosis and management occurring in a non-endemic setting, and recent surveillance frameworks emphasize the need for minimum epidemiological data, including notification location, residence and most likely location of infection (Arisco et al., 2022; Safarpour et al., 2025). In China, previous molecular and genomic studies have shown that parasite genetic data can support geographic assignment of imported *P. falciparum* cases, confirmation of the absence of local transmission, and investigation of possible non-travel or locally linked infections in the post-elimination era (Liu et al., 2020; Luo et al., 2025).

Imported-case and traveler-based surveillance also has broader value beyond China. Analysis of imported malaria to non-endemic countries showed that international malaria importation is highly structured by travel and migration routes, with West Africa accounting for a large proportion of imported malaria cases reported by non-endemic countries with travel-destination data (Safarpour et al., 2025). Reviews of imported malaria further show that detected cases are shaped by traveler type, immunity, travel history, duration of exposure, clinical presentation, and healthcare access (Chang et al., 2019; Tessema et al., 2019). For malaria elimination programs, molecular and genomic information from imported cases can therefore contribute to source attribution, importation-route monitoring, drug-resistance surveillance and reintroduction-risk assessment (Preston et al., 2014). At the same time, these data are generated through a movement- and detection-defined pathway: parasites are observed only after infection in an endemic region, travel, symptom onset or testing, clinical diagnosis, reporting and sample processing (Mayor et al., 2023).

This pathway creates an important interpretive issue. Imported-case genomes are valuable surveillance data, but they are not necessarily random or directly interchangeable samples of parasite populations circulating in the presumed regions of origin (Wohl et al., 2023). In this study, we use the term surveillance-defined sampling window to describe the process through which infections become represented in an imported-malaria genomic dataset. This sampling window includes epidemiological and healthcare processes such as travel, illness or testing, diagnosis, reporting, and specimen availability. It differs from direct endemic-setting sampling, which captures parasites through local clinical, community, or research activities. The term does not imply that any observed genomic difference can be attributed uniquely to surveillance pathway, because geographic composition, sampling period, sequencing characteristics, variant-calling procedures, and within-host infection complexity may also affect cross-dataset comparisons.

This concern is consistent with recent guidance on molecular surveillance, which emphasizes that the design of molecular surveillance must begin by defining the target population, sampling technique, sampling period, sampling frequency and sample size required to draw valid and unbiased conclusions (Nsanzabana, 2019; Mensah et al., 2022; Mayor et al., 2023). Empirical work using antenatal-care users as a genomic malaria surveillance population illustrates the same principle: a defined sampling channel can provide valuable signals about transmission and intervention impact, but it represents a particular surveillance population rather than the entire parasite population (Brokhattingen et al., 2024). Similar concerns apply when imported genomes are compared with public reference panels. Resources such like *Pf8* have transformed malaria population genomics by providing large-scale views of *P. falciparum* variation, but public reference datasets remain products of specific sampling locations, collection periods, study designs, sequencing procedures and variant filters (Abdel Hamid et al., 2025).

Population-genomic interpretation is also sensitive to sample composition and analytical framing. Structure plots are widely used alongside PCA as descriptive summaries of genetic data, but methodological work cautions against reading inferred components literally as fixed ancestral populations or discrete biological entities (Lawson et al., 2018). Sample selection bias can affect the accuracy of both model-based ancestry inference and structure recovery (Shringarpure and Xing, 2014). These issues are especially relevant for *P. falciparum*, where infections may be polyclonal and where highly polymorphic, subtelomeric or recombinant antigenic loci can complicate SNP discovery, mapping, diversity estimation and evolutionary interpretation (Su et al., 2019). Consequently, differences in PCA, phylogenetic reconstruction, diversity, differentiation, or allele-frequency-spectrum summaries between imported and endemic-reference datasets may reflect a mixture of parasite biology, sampling framework, reference-panel composition, data quality, and analytical choices.

Here, we analyzed West Africa-associated *P. falciparum* genomes detected through China’s post-elimination imported-malaria surveillance system and compared them with public and locally collected West African reference isolates. This focus reflects the epidemiology of imported malaria in China, where most imported *P. falciparum* cases have been acquired in African countries and several West and Central African countries are major reported sources (Li et al., 2026). West Africa-associated infections therefore provide a relevant case study for examining how imported-case genomic surveillance captures parasite diversity entering a malaria-free country.

We evaluated within-host infection complexity, identity-by-descent, population structure, nucleotide diversity, allele-frequency differentiation, and Tajima’s D. To assess the sensitivity of these comparisons to reference-panel composition, genotype filtering, and within-host complexity, we used country-balanced reference subsampling, geographically restricted and quality-controlled population-structure analyses, and increasingly stringent F_WS_ restricted subsets of imported isolates. Our aim was not to infer a surveillance pathway-specific biological effect or to treat imported genomes as direct proxies for source-population diversity. Instead, we examined which population-genomic patterns were retained across these analytical conditions and which were sensitive to dataset construction. This framework clarifies both the utility and the interpretive limits of imported-case genomic data for post-elimination malaria surveillance.

## 2 Methods

### 2.1 Study population and genome sequencing

China-imported *P. falciparum* cases were identified through the post-elimination malaria surveillance system in China. Patients were diagnosed at local clinical facilities and reported through local Centers for Disease Control and Prevention (CDC) networks. Blood specimens and associated epidemiological records were subsequently submitted to the national parasite-resource system. The present study used de-identified archived specimens and associated data obtained through this resource framework.

Our previous study (Chen et al., 2017) showed that direct sequencing will not affect the data integrity. DNA was extracted using the DNeasy Blood & Tissue Kit (QIAGEN, UK). Libraries were prepared using the TruSeq kit (Illumina, USA) and sequenced on an Illumina X-10 platform, generating 41–212 million 150-bp paired-end reads per sample. Reads were quality filtered using Trimmomatic v3.0 (Bolger et al., 2014) and aligned to the *P. falciparum* 3D7 reference genome using BWA (Li and Durbin, 2010). Variant calling was conducted using the GATK4 workflow (Mckenna et al., 2010). Detailed sample metadata, sequencing metrics, and analysis-specific inclusion status are provided in **Supplementary Table S1**.

Public West African reference genomes were obtained from the *Pf3k* project database (https://www.malariagen.net/project/pf3k/). Additional locally collected isolates from Togo and Sierra Leone were included as an independently generated endemic comparison dataset. Dataset-specific selection, quality-control criteria, and sensitivity analyses are described below.

### 2.2 Dataset construction and sensitivity analyses

To assess the sensitivity of cross-dataset comparisons to reference-panel composition, genotype quality, and within-host infection complexity, we constructed four additional reference and datasets (R2, D1, D2a, and D2b) in addition to the original analysis in **Table 1**. Across the revised datasets, only biallelic SNPs were retained; indels and multiallelic variants were excluded. Unless otherwise specified, samples were required to have a parasite-mapping proportion of at least 90%, and sites and genotypes were required to meet missingness thresholds of 0.02. Variant-quality filtering followed the criteria applied to the *Pf3K* VCF, including the corresponding GQ, QUAL, and VQSLOD filters. The complete filtering workflow is provided in **Supplementary Methods** note.

**Table 1:** Analytical datasets used to assess sensitivity to reference-panel composition, filtering, and within-host complexity.

Dataset	Imported isolates	Reference composition	SNP processing	Analyses performed	Analytical purpose
Original	31	original reference	Original pipeline; DP ≥ 3	Original PCA/tree/Structure; π, F_ST_, Tajima’s D	Initial cross-dataset comparison
R2	31	13 countries; 30 isolates/country; 20 random replicates	Original filtering	PCA	Sensitivity to country-level reference sample imbalance
D1	31 QC-passed	5 West African countries; 30 isolates/country; plus local comparison samples	DP ≥ 5; QC; core genome; LD pruning	PCA, tree, STRUCTURE	Geographically restricted and quality-controlled population-structure comparison
D2a	4, FWS>0.95	Same as D1	Same core-genome, LD-pruned SNP set as D1	PCA, tree	Qualitative near-monoclonal placement sensitivity
D2b	9, FWS>0.90	150 Pf3K isolates from the same five countries, 30/country	DP ≥ 5; QC; no LD pruning	π, F_ST_ ​, Tajima’s D	Sensitivity of diversity, differentiation, and allele-frequency-spectrum summaries

R2 evaluated the effect of unequal reference-country sample sizes. A maximum of 30 *Pf3K* isolates was randomly selected from each of 13 countries, and the procedure was repeated 20 times using the filtering approach of the original analysis. PCA was performed for each replicate.

D1 was used for the revised population-structure analysis. It included 31 China-imported isolates, 150 *Pf3K* isolates randomly selected from 5 West African countries (30 isolates per country), 7 locally collected Togo isolates (Kassegne et al., 2020), 4 locally collected Sierra Leone isolates (Wang et al., 2024), and 9 previously reported Ghana-associated imported isolates from Guangxi, China (Shi et al., 2021). Genotypes were filtered at DP ≥ 5, restricted to the core genome, and pruned for linkage disequilibrium using PLINK before PCA, phylogenetic reconstruction, and STRUCTURE analysis.

D2a assessed the compatibility of population placement after a stringent restriction for within-host infection complexity. It included four imported isolates with F_WS_>0.95 and 150 *Pf3K* reference isolates randomly selected from 5 West African countries (30 isolates per country). The dataset used the same DP ≥ 5, core-genome, and LD-pruned SNP framework as D1. PCA and phylogenetic reconstruction were performed as a qualitative near-monoclonal sensitivity analysis and are presented in **Supplementary Figure S1**.

D2b assessed the sensitivity of nucleotide diversity, gene-level F_ST_, and Tajima’s D to stricter depth filtering and reduced within-host complexity. It included 9 imported isolates with F_WS_>0.90 and the same 150-isolate *Pf3K* reference panel. Genotypes were filtered at DP ≥ 5 and restricted to the core genome but were not LD pruned because these statistics depend on the distribution of segregating sites. D2b results were compared with those of the original dataset to identify summaries that were retained or altered under the revised filtering and sample-restriction scheme.

These analyses addressed several sources of non-comparability but did not remove differences in sampling period or historical data generation between datasets. The resulting comparisons were therefore interpreted as sensitivity analyses of datasets constructed under distinct sampling and analytical frameworks.

### 2.3 Within-host infection complexity and relatedness analyses

Within-host infection complexity was assessed using the within-host fixation index, F_WS_​. For the China-imported isolates and the previously generated comparison isolates included in this study, F_WS_ ​was calculated from high-quality biallelic SNPs using the moimix (Lee and Bahlo, 2016). For *Pf3K* reference isolates, F_WS_ were obtained from the corresponding MalariaGEN sample metadata. Higher F_WS_ values indicate lower within-host diversity and a greater predominance of a single parasite genotype.

The F_WS_>0.90 and F_WS_>0.95 thresholds were applied to the 31 newly sequenced China-imported isolates only, to define the D2b and D2a sensitivity subsets, respectively. These thresholds were used to evaluate the potential influence of within-host infection complexity on downstream analyses and were not interpreted as absolute biological definitions of monoclonal infection.

Pairwise identity-by-descent (IBD) sharing among the China-imported isolates was estimated using hmmIBD (Schaffner et al., 2018) with D1 set after core area and LD-pruned filtering. IBD was used to describe recent genetic relatedness among imported isolates and was not used as the primary basis for cross-dataset inference.

### 2.4 Population structure and phylogenetic reconstruction

Population structure was evaluated using principal component analysis, maximum-likelihood phylogenetic reconstruction, and STRUCTURE analysis (Pritchard et al., 2000). R2 was used to assess the sensitivity of PCA results to country-level imbalance in the reference panel. PCA was repeated across 20 country-balanced random subsampling replicates, as described in Section 2.2.

The primary restricted population-structure analysis was conducted using the D1 dataset. D1 included 201 isolates and was filtered using a minimum genotype depth of DP ≥ 5, a parasite-mapping proportion of at least 90%, and maximum missingness thresholds of 2% at both the sample and site levels. Only biallelic SNPs in the defined core genome were retained. Variants were pruned for linkage disequilibrium using PLINK2 (Chang et al., 2015) before PCA, phylogenetic reconstruction, and STRUCTURE analysis. The final D1 SNP set contained 272,300 SNPs after MAF 0.05 filtered.

PCA was performed using pcadapt (Luu et al., 2017) on the LD-pruned D1 SNP set. Maximum-likelihood phylogenetic trees were inferred from the same SNP set using RAxML-NG (Kozlov et al., 2019). Node support was evaluated using 1,000 bootstrap replicates. STRUCTURE analysis was performed on the D1 SNP set. The most informative K values were selected based on cross-validation error and biological interpretability (Earl and Vonholdt, 2012).

D2a used the same DP ≥ 5, core-genome, and LD-pruned framework as D1, but included only 4 isolates with F_WS_>0.95 and 150 *Pf3K* reference isolates. PCA and phylogenetic reconstruction were repeated to assess whether the broad placement of this near-monoclonal imported subset was compatible with the corresponding D1 and original analyses. Given the limited number of imported isolates in D2a, this analysis was interpreted qualitatively and was not used for country-level assignment or formal population-level inference.

### 2.5 Nucleotide diversity and allele-frequency differentiation analyses

Nucleotide diversity (π) and F_ST_ were calculated from the D2b dataset to evaluate the sensitivity of diversity and differentiation estimates to stricter genotype filtering and reduced within-host infection complexity. D2b genotypes were filtered using a minimum depth of DP ≥5, mapping proportion of at least 90%, and maximum missingness thresholds of 2% at both the sample and site levels. Only high-quality biallelic SNPs were retained, and indels and multiallelic variants were excluded. Variants were restricted to the core genome but were not pruned for linkage disequilibrium, because both π\piπ and gene-level F_ST_ depend on the observed distribution of segregating sites across genes.

Nucleotide diversity was calculated for each annotated gene using Arlequin3.5 (Excoffier and Lischer, 2010), and was summarized across the genomic background and predefined gene categories. Pairwise gene-level F_ST_ was estimated between China-imported isolates and the *Pf3K* reference panel on SNPs shared between the two datasets. The D2b results were compared with the original DP ≥ 3 analysis, which included 31 China-imported isolates, to determine whether central diversity and differentiation summaries were retained after the revised filtering and F_WS_​-based sample restriction.

For the F_ST_ comparison, we summarized the signed paired differences using the mean, median, quartiles, and the numbers of genes with positive, negative, zero, or non-estimable differences. Gene-level results were interpreted as sensitivity summaries and were not used to identify selection candidates or to infer population-specific adaptation.

### 2.6 Allele-frequency spectrum analyses

Tajima’s DDD was calculated for each annotated gene to summarize allele-frequency-spectrum patterns in the China-imported and *Pf3K* reference datasets. Analyses were conducted using the D2b dataset. D2b genotypes were filtered using a minimum depth of DP ≥ 5, mapping proportion of at least 90%, and maximum sample and site-level missingness thresholds of 2%. Only biallelic SNPs in the core genome were retained; indels and multiallelic variants were excluded. LD pruning was not applied because Tajima’s D depends on the observed distribution of segregating sites and allele frequencies.

D2b estimates were compared with those from the original analysis, which used 31 China-imported isolates and the original DP ≥ 3 filtering framework. This comparison was used to assess the sensitivity of Tajima’s D summaries to stricter genotype filtering, the resulting variant set, and restriction of imported isolates by F_WS_. Tajima’s D was interpreted as a descriptive summary of the observed allele-frequency spectrum and was not used as stand-alone evidence of selection, demographic history, or surveillance-specific biological change.

## 3 Result

### 3.1 Geographic background and surveillance frameworks of the analyzed genomes

We first summarized the geographic origins and sampling pathways of the analyzed genomes (**Figure 1**). All datasets were associated with West African transmission settings, but they were obtained through different sampling frameworks. The endemic/reference datasets included publicly available *Pf3K* genomes and locally collected isolates from Togo and Sierra Leone. These samples were collected within endemic transmission settings through local clinical, community, or research-based sampling. The imported-surveillance dataset comprised malaria cases detected through China’s post-elimination surveillance system. These infections were acquired in malaria-endemic settings but became observable only after international travel, clinical presentation or testing, diagnosis, reporting, and specimen collection in China.

**Figure 1:**
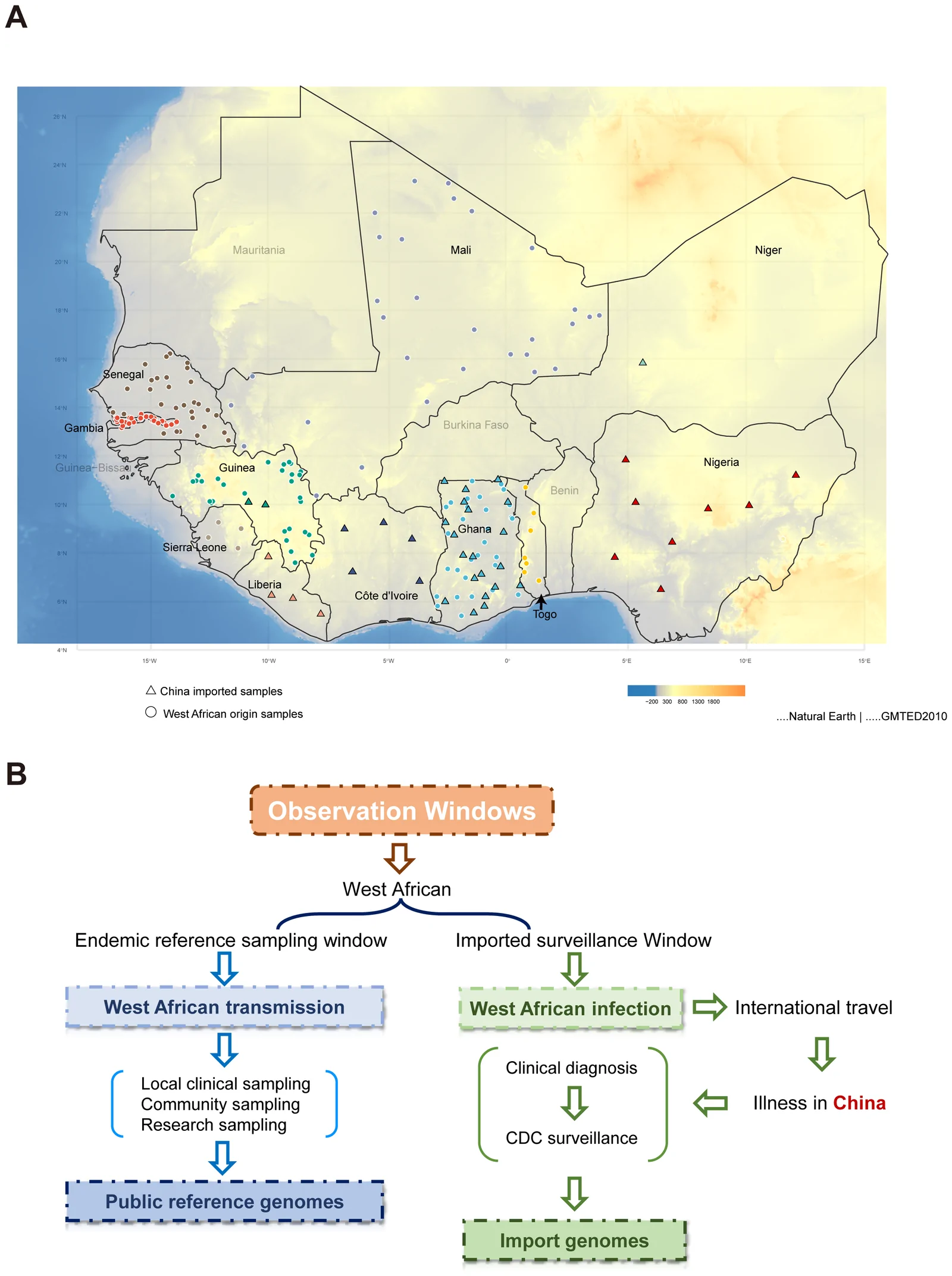
Geographic background and surveillance frameworks of West Africa-associated *P. falciparum* genomes. **(A)** Geographic distribution of the West African reference/comparison datasets and China-imported malaria isolates included in this study. Reference/comparison datasets comprised publicly available *Pf3K* genomes and previously generated locally collected isolates from Togo and Sierra Leone. China imported isolates were detected through the post-elimination malaria surveillance system in China. **(B)** Conceptual representation of the sampling frameworks. Endemic/reference datasets were assembled from parasites sampled within West African transmission settings, whereas imported isolates were observed after infection acquisition, international travel, clinical presentation or testing, diagnosis, reporting, and specimen availability in China. The schematic illustrates differences in sampling frames and does not imply that any observed genomic difference is attributable solely to surveillance pathway.

Thus, the imported isolates represent a surveillance-defined subset of infections associated with West Africa, whereas the reference datasets represent samples collected through endemic-setting surveillance or research activities. The two sampling frameworks are not equivalent and may differ in geographic composition, collection period, sequencing characteristics, and data-processing history. We therefore did not attribute cross-dataset genomic differences to surveillance pathway alone. Instead, we used geographically restricted, country-balanced, quality-controlled, and F_WS_ analyses to assess the sensitivity of the subsequent population-structure, diversity, differentiation, and allele-frequency-spectrum results.

### 3.2 Within-host diversity and genetic relatedness of imported malaria isolates

We first assessed within-host infection complexity among the 31 China-imported isolates using F_WS_ (**Figure 2A**; **Supplementary Table S1**). The values varied across isolates, indicating heterogeneous within-host parasite diversity in the imported dataset. 9 isolates had F_WS_>0.90, and 4 had F_WS_>0.95. These two subsets were subsequently used for the D2b and D2a sensitivity analyses, respectively.

**Figure 2:**
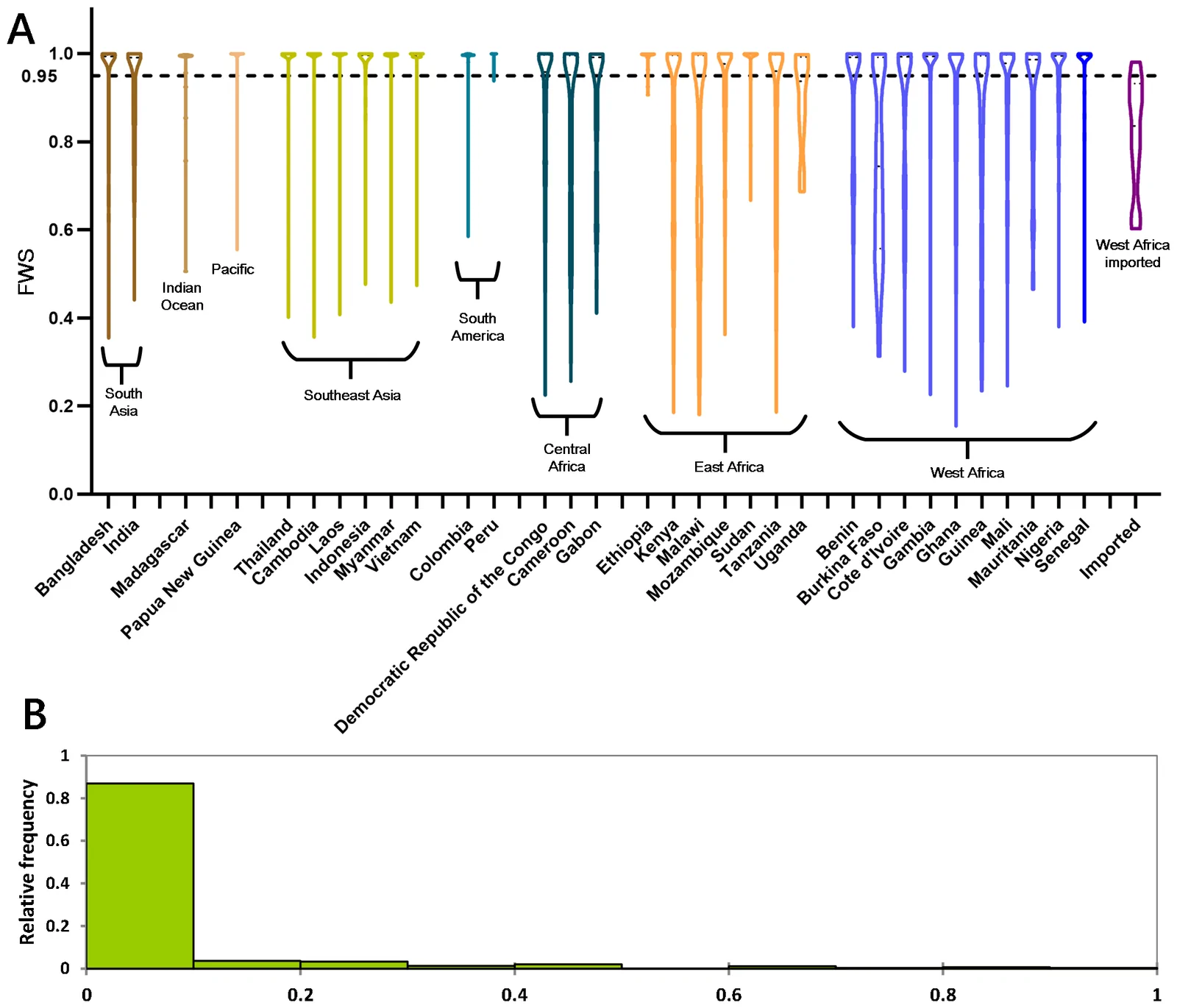
Within-host infection complexity and pairwise relatedness among China-imported *P. falciparum* isolates. **(A)** Distribution of F_WS_ values among China-imported isolates and reference datasets. Higher F_WS_ values indicate lower within-host diversity. Dashed lines indicate the F_WS_>0.90 and F_WS_>0.95 thresholds used to define the D2b and D2a sensitivity subsets, respectively. **(B)** Pairwise IBD sharing among China-imported isolates. IBD estimates are presented as descriptive summaries of within-dataset relatedness.

Pairwise IBD sharing was estimated among the imported isolates to describe genetic relatedness within the dataset (**Figure 2B**). Most pairwise comparisons showed limited IBD sharing, consistent with low levels of recent relatedness among many of the sampled infections. However, because imported samples included infections with variable within-host complexity and were represented using dominant-allele calls, IBD results were interpreted as descriptive and were not used as the primary basis for cross-dataset inference.

Together, the F_WS_ results show that the full imported dataset contained infections with differing levels of within-host complexity. We therefore evaluated subsequent population-genetic results using the full quality-controlled dataset and progressively stricter F_WS_​ restricted subsets.

### 3.3 Population structure under restricted and quality-controlled comparisons

We first examined whether unequal representation of reference countries could influence the placement of China-imported isolates. Across 20 country-balanced R2 subsampling replicates, the broad PCA pattern was qualitatively similar, indicating that the observed placement was not dependent on a single selection of *Pf3K* reference isolates (**Supplementary Figure S1**; **Supplementary Table S2**).

We then performed the primary population-structure analysis using the D1 dataset, which included 31 China-imported isolates, 150 *Pf3K* reference isolates from five West African countries, 7 locally collected Togo isolates, 4 locally collected Sierra Leone isolates, and 9 previously reported Ghana-associated imported isolates from Guangxi, China (total n=201; **Figures 3A, B, D**). In the D1 PCA, the first two principal components explained 28.15% and 3.12% of the variance, respectively. The China-imported isolates occupied a distribution separated from the main *Pf3K* reference-sample cloud along the first two principal components. In contrast, the 5 *Pf3K* country groups showed substantial overlap in the PCA space. The locally collected Togo and Sierra Leone isolates were positioned within or close to the *Pf3K* West African distribution, whereas the previously reported Ghana-associated imported isolates provided an additional imported comparison group.

**Figure 3:**
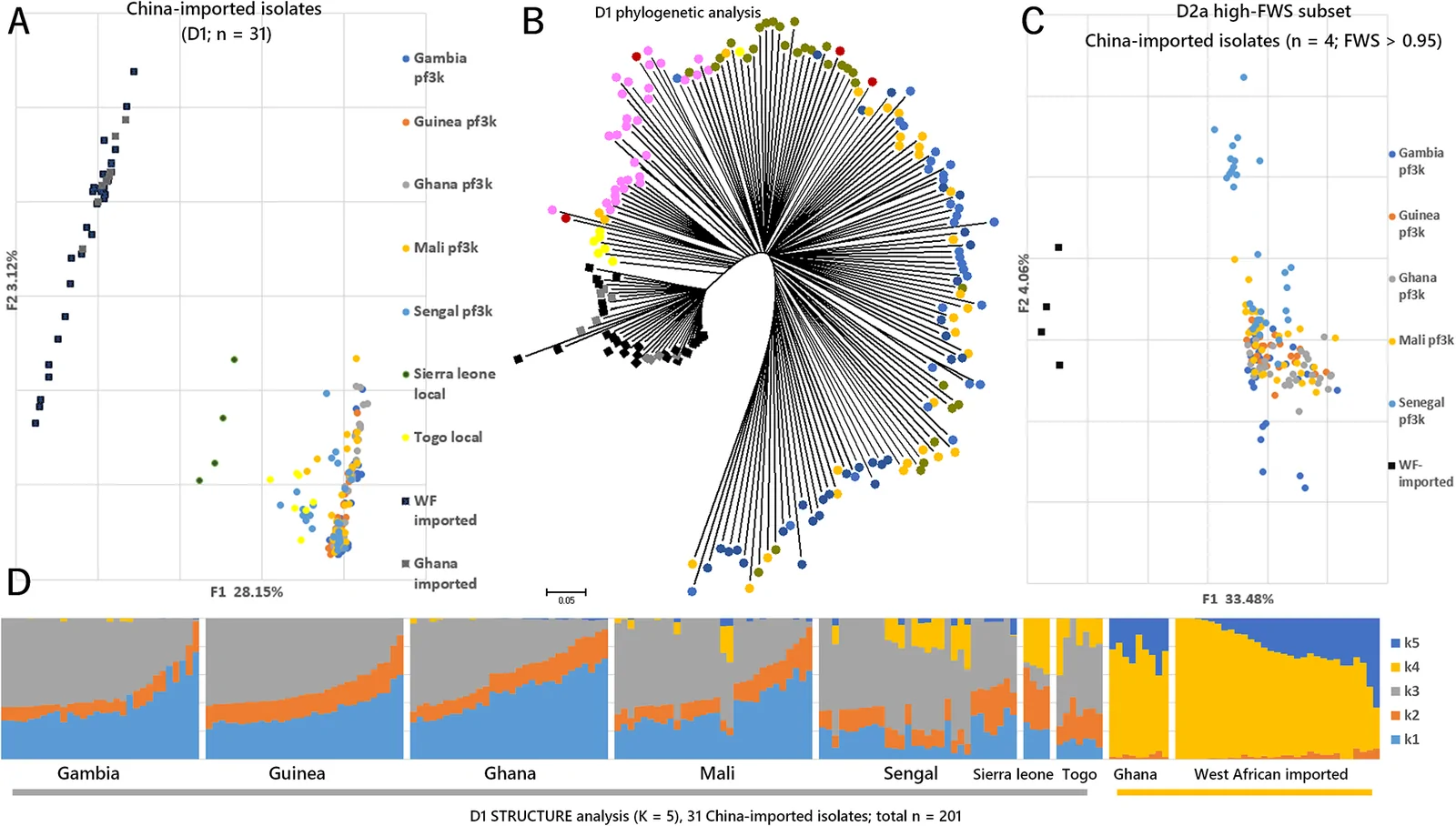
Population structure of China-imported and West African comparison isolates under the D1 framework, with a D2a high F_WS_ sensitivity analysis. **(A, B, D)** D1 analyses of 201 isolates, including 31 China-imported isolates, 150 *Pf3K* reference isolates from Gambia, Guinea, Ghana, Mali, and Senegal (30 isolates per country), 7 locally collected Togo isolates, 4 locally collected Sierra Leone isolates, and 9 previously reported Ghana-associated imported isolates from Guangxi, China. D1 analyses used DP ≥ 5, quality-controlled, core-genome, LD-pruned biallelic SNPs. **(A)** PCA of the D1 dataset. PC1 and PC2 explained 28.15% and 3.12% of the variance, respectively. **(B)** Maximum-likelihood phylogenetic tree of the D1 dataset. The scale bar indicates substitutions per site; node support was evaluated using 1,000 bootstrap replicates. **(C)** D2a PCA including 150 *Pf3K* West African reference isolates and 4 China-imported isolates with F_WS_>0.95 (n=154). D2a used the same DP ≥ 5, core-genome, and LD-pruned SNP framework as D1. Togo, Sierra Leone, and the previously reported Ghana-associated imported comparison isolates were not included in D2a. PC1 and PC2 explained 33% and 4% of the variance, respectively. **(D)** STRUCTURE analysis of the D1 dataset at K = 5. Colors denote model-based ancestry components and should not be interpreted as discrete ancestral populations.

The maximum-likelihood phylogenetic tree showed that the China-imported isolates were placed in a restricted region of the overall topology rather than being uniformly distributed among the *Pf3K* reference isolates **Figure 3B**). The STRUCTURE analysis at K = 5 likewise showed that the China-imported isolates had a component profile distinct from the predominant profiles in the selected *Pf3K* reference panel (**Figure 3D**). These model components were used as descriptive summaries of the D1 data and were not interpreted as discrete ancestral populations.

To evaluate whether the observed population placement was strongly dependent on within-host infection complexity, we repeated PCA using the D2a subset of four China-imported isolates with FWS>0.95 and the 150 isolate *Pf3K* West African reference panel (**Figure 3C**). The 4 high F_WS_ isolates remained separated from the central *Pf3K* reference cloud and occupied a position compatible with the broader distribution of China-imported isolates in the D1 PCA. Because D2a contained only 4 imported isolates, this result was treated as a qualitative sensitivity assessment and was not used for country-level assignment or formal population-level inference.

Together, these analyses show that the broad population placement of the China-imported isolates was retained after country balanced reference subsampling, more stringent quality filtering, and restriction to a high-F_WS_ ​subset. However, the imported and reference datasets differed in sampling period, composition, and historical data generation. We therefore interpret these results as evidence that China-imported surveillance isolates were not fully interchangeable with the selected West African comparison datasets, rather than evidence that surveillance pathway alone caused the observed structure.

### 3.4 Sensitivity of nucleotide diversity and gene-level F_ST_ estimates to the D2b analysis

We compared nucleotide-diversity and gene-level differentiation estimates between the original analysis and the D2b sensitivity analysis. In both settings, China-imported isolates were compared with a *Pf3K* reference panel comprising 150 isolates from 5 West African countries, with 30 isolates randomly selected per country. The original analysis included 31 China-imported isolates, whereas D2b included only 9 isolates with F_WS_>0.90 and applied the revised DP ≥ 5 and quality-control filters.

Gene-level F_ST_ values were heterogeneously distributed across the 14 chromosomes in both analyses (**Figures 4A, B**). Drug-resistance-associated genes and selected antigen-gene categories were distributed across a broad range of F_ST_ values in both settings. Central F_ST_ summaries changed little between the original and D2b analyses. Among genes with paired estimable values, the mean and median differences, calculated as F_ST_ D2b−F_ST_ original, were −0.0024 and −0.00014, respectively. However, gene-specific estimates changed in both directions, while 2,794 genes had higher and 2,506 had lower F_ST_ values in D2b. The interquartile range of paired differences was −0.075 to 0.078 (IQR, 0.153; **Supplementary Figure S3**), indicating heterogeneous gene-level sensitivity to the revised filtering and sample-restriction scheme.

**Figure 4:**
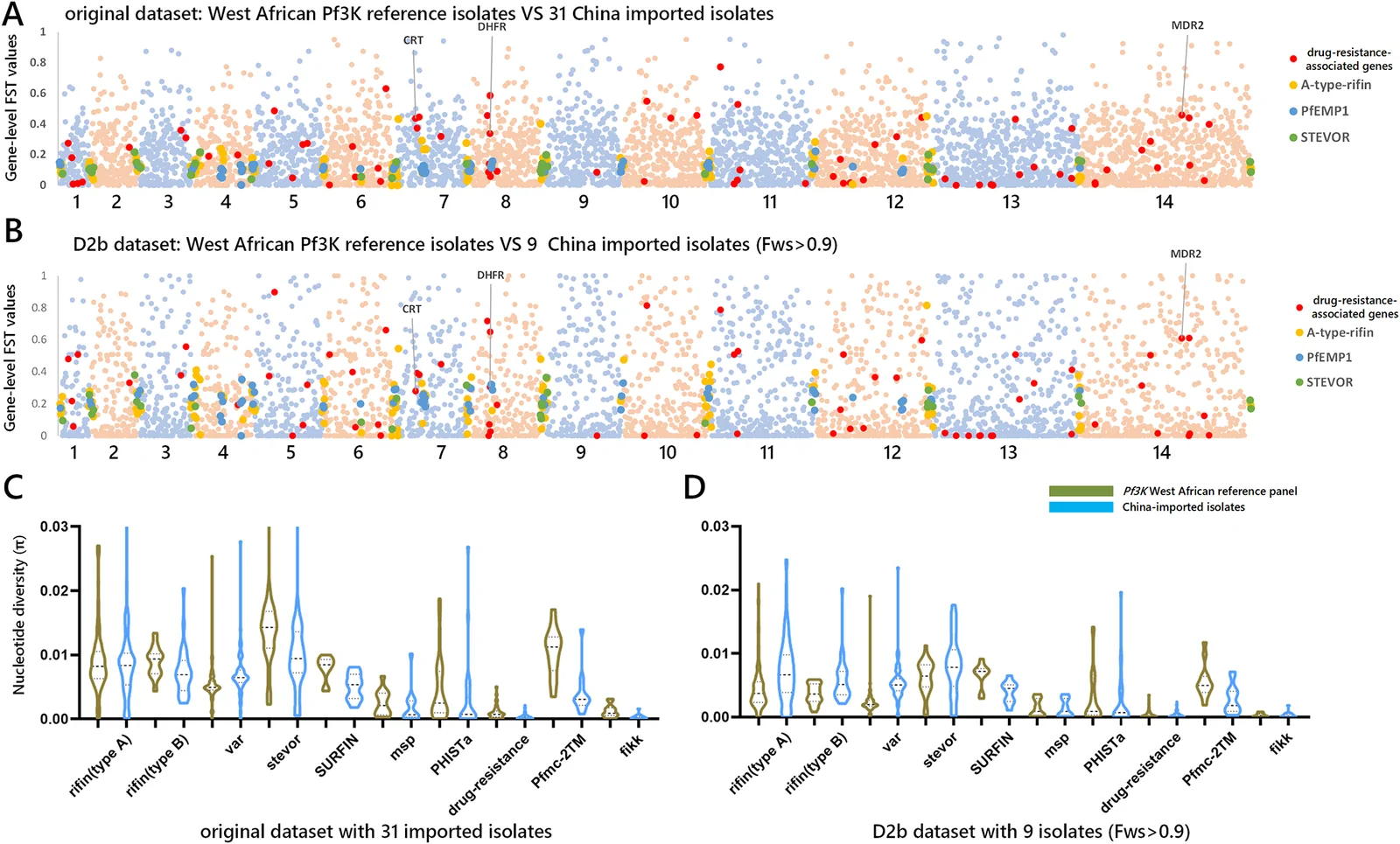
Sensitivity of gene-level nucleotide diversity and F_ST_ estimates to the D2b analysis. China-imported isolates were compared with a *Pf3K* reference panel comprising 150 isolates randomly selected from 5 West African countries (30 isolates per country). The original analysis included 31 China-imported isolates. D2b included nine China-imported isolates with FWS>0.90 and applied DP ≥ 5, quality-control, and core-genome filters without LD pruning. **(A, B)** Gene-level F_ST_ values across the 14 chromosomes in the original analysis **(A)** and D2b analysis **(B)**. Colored points identify pre-specified gene categories; all other genes are shown as background points. **(C, D)** Gene-level nucleotide diversity (π) distributions in West African *Pf3K* reference isolates and China-imported isolates in the original analysis **(C)** and D2b analysis **(D)**. Dashed horizontal lines indicate median values.

Gene-level nucleotide-diversity distributions also differed between the *Pf3K* reference panel and China-imported isolates in both analyses (**Figures 4C, D**). The contrast remained visible after restriction to the nine high-F_WS_ imported isolates, although the absolute π\piπ distributions varied across predefined gene categories. In particular, the distributions of π\piπ in the variant-surface-antigen categories, including var, rifin, and STEVOR, changed between the original and D2b analyses. These category-specific estimates were interpreted cautiously because of their high polymorphism and limited short-read mappability.

D2b simultaneously changed genotype-depth filtering, quality-control restrictions, the imported-sample subset, and the resulting variant set. Therefore, the observed changes cannot be assigned to a single filtering parameter or interpreted as evidence that either dataset is intrinsically biased. We retained the broad cross-dataset diversity and differentiation patterns as descriptive comparisons but did not use individual gene-level F_ST_ values or category-specific extremes as evidence of selection or population-specific adaptation.

### 3.5 Sensitivity of allele-frequency-spectrum summaries to filtering and sample restriction

We compared gene-level Tajima’s D values obtained in the original analysis and in the D2b sensitivity analysis (**Figure 5**). In both settings, China-imported isolates were compared with the *Pf3K* West African reference panel. The original analysis included 31 China-imported isolates, whereas D2b included nine isolates with F_WS_>0.90 and applied the revised DP ≥ 5, quality-control, and core-genome filters without LD pruning.

**Figure 5:**
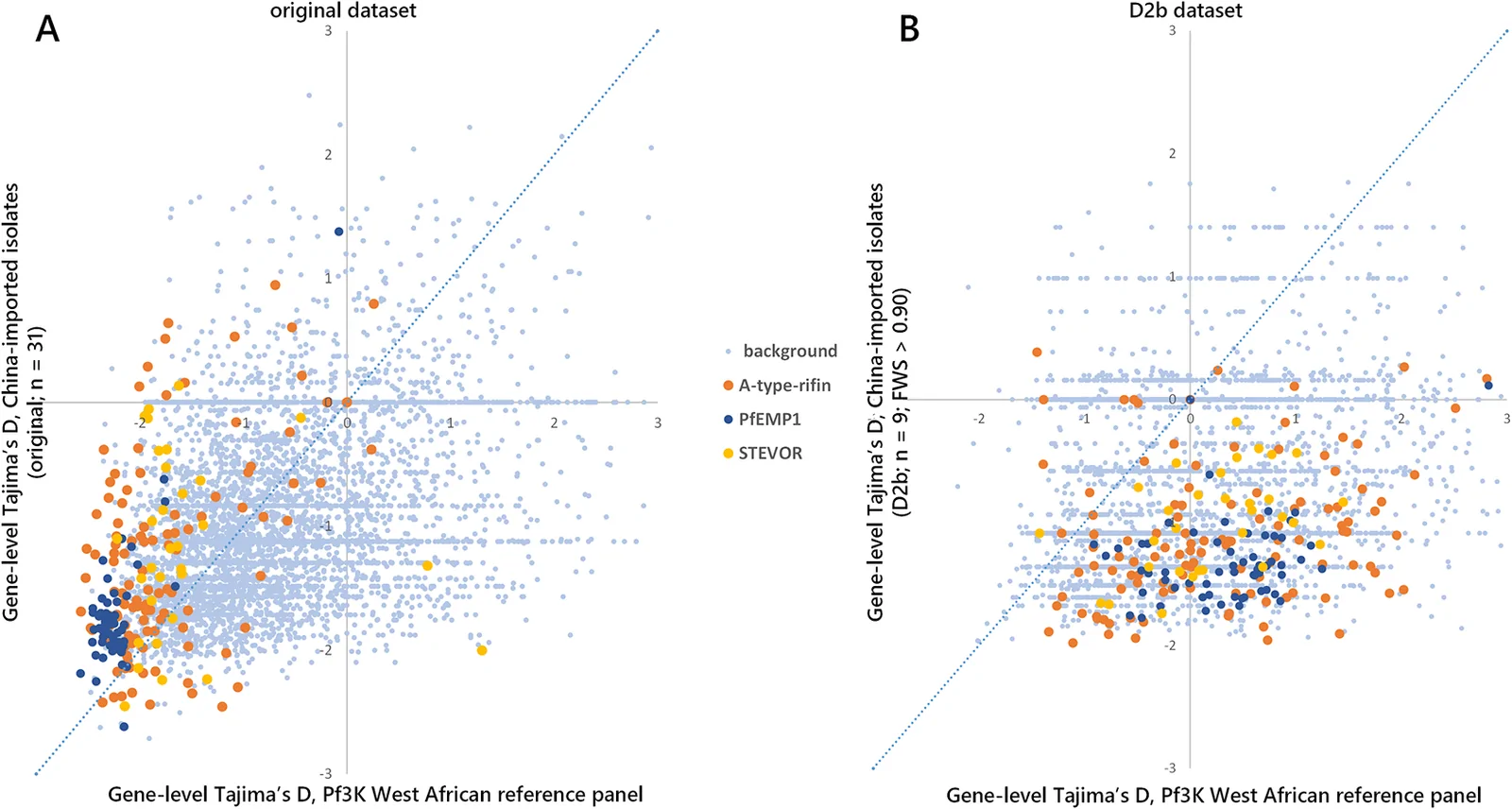
Sensitivity of gene-level Tajima’s D estimates to the D2b filtering and F_WS_ restricted analysis. Gene-level Tajima’s D values in China-imported isolates are plotted against those in the *Pf3K* West African reference panel. **(A)** Original analysis including 31 China-imported isolates. **(B)** D2b analysis including nine China-imported isolates with F_WS_>0.90, using DP ≥ 5, quality-control, and core-genome filters without LD pruning. Colored points indicate selected antigen-gene categories; all other genes are shown as background points. Tajima’s D was used as a descriptive allele-frequency-spectrum summary and was not interpreted as stand-alone evidence of selection or demographic history.

In the original analysis, mean gene-level Tajima’s D was -1.8 in reference panel and -1.3 in the China-imported isolates. In D2b, the corresponding mean values were 0.31 and -1.13, respectively. Thus, the mean Tajima’s D of the imported subset remained negative after F_WS_ restriction, whereas the *Pf3K* reference-panel summary shifted markedly toward positive values. Consequently, the relative imported-versus-reference contrast observed in the original analysis was not retained in D2b.

A similar change was observed for the selected antigen-gene categories. In the original analysis, the mean Tajima’s D values for these categories were -0.60 in the *Pf3K* reference panel and -0.80 in the China-imported isolates. In D2b, the corresponding values were -0.04 and -0.50, respectively. These values indicate that the absolute Tajima’s D estimates and their contrast were sensitive to the revised filtering and sample restriction scheme.

Because D2b simultaneously changed DP filtering, QC restrictions, the imported-sample subset, and the resulting SNP set, these changes cannot be attributed to a single analytical factor. We therefore interpret Tajima’s D as a descriptive summary of the observed allele-frequency spectrum and do not use the differences as stand-alone evidence of selection, demographic history, or a surveillance-pathway-specific biological effect.

## 4 Discussion

Genomic surveillance is now widely regarded as an important component of infectious disease surveillance and malaria elimination programs, rather than a purely research-oriented tool. In malaria, this programmatic rationale has been developed further in the malaria genomic literatures which argue that genomic epidemiology can help monitor parasite population change, transmission intensity, drug resistance, gene flow and residual transmission as countries move toward elimination (Neafsey and Volkman, 2017; Wesolowski et al., 2018). More recent implementation-focused studies have also shown that flexible sequencing strategies, including targeted nanopore sequencing, can generate policy-relevant information on antimalarial resistance, diagnostic escape and vaccine-relevant variation, indicating that malaria genomic surveillance is moving from concept toward practical public-health application (Nsanzabana, 2019; De Cesare et al., 2024). This rationale is especially relevant in China’s post-elimination setting, where the principal challenge is no longer sustained indigenous transmission but the detection and management of imported malaria (Li and Zhigui, 2024; Zhu et al., 2024). Imported malaria has itself been formalized as a surveillance category, with recent work emphasizing the need for minimum epidemiological data to classify imported infections and support elimination programs (Arisco et al., 2022). WGS has been applied to investigate non-travel malaria cases potentially linked to imported infections (Luo et al., 2025). These studies provide the operational context for the present work: in a post-elimination country, genomic data can complement routine epidemiological information for case classification, broad source assessment, and investigation of possible onward transmission, particularly when travel history or conventional epidemiological information is incomplete.

Imported-case and traveler-based surveillance also has broader public-health value beyond China. Reviews on detecting imported malaria infections in endemic settings emphasized that human mobility can move parasites across regions and that molecular or genomic tools, combined with epidemiological and traveler information, can improve detection and characterization of imported infections (Mischlinger et al., 2020; Safarpour et al., 2025). This means that imported malaria genomes are not peripheral data; they directly represent the parasite diversity that enters a country through travel, labor migration or other forms of cross-border movement, and they are therefore highly relevant for source attribution, monitoring importation routes, updating drug-resistance information and assessing reintroduction risk. However, imported infections are defined through movement and detection rather than through random sampling in the source population. Imported-case genomes are valuable surveillance data, but they should not automatically be treated as unbiased population samples from the countries or regions where infection was acquired. Their interpretation should also account for reference-panel composition, sample quality, within-host complexity, and analytical processing.

Against this background, our study provides an empirical example of the interpretive challenges involved in comparing genomes obtained through imported-malaria surveillance with parasites sampled in endemic settings. The China-imported, West Africa-associated isolates were not intended to represent a random cross-section of parasite populations circulating in West Africa. Rather, they represent infections observed through travel, clinical presentation or testing, diagnosis, reporting, and specimen availability in China. The additional sensitivity analyses strengthened the interpretation of this comparison. Population-structure patterns were retained after country-balanced reference subsampling, geographically restricted comparison, stricter quality filtering, and restriction to high-F_WS_​ imported subsets. In contrast, some diversity and allele-frequency-spectrum summaries, particularly gene-level F_ST_ estimates and Tajima’s D, were sensitive to the revised filtering and sample-restriction scheme. These results indicate that imported isolates were not fully interchangeable with the selected West African comparison datasets, while also showing that the magnitude and direction of some cross-dataset summaries depend on dataset construction. We therefore interpret the observed differences as features of comparisons between surveillance-derived and endemic-reference datasets, rather than as effects attributable uniquely to surveillance pathway. Geographic composition, sampling period, sequencing characteristics, reference-panel construction, and analytical filtering may all contribute. This framework does not reduce the value of imported-case genomes; instead, it defines their appropriate use as surveillance-derived data for regional population placement and monitoring, rather than as unqualified proxies for source-population structure.

These findings support a cautious interpretation of imported-case genomic data. Imported malaria in non-endemic countries is defined by acquisition in an endemic area followed by travel, clinical manifestation or testing, diagnosis, and management in a non-endemic setting; it is therefore a movement- and detection-defined category rather than a random sampling category (Mischlinger et al., 2020). Imported-case genomes remain valuable for regional population placement, monitoring importation routes, updating drug-resistance information, and assessing reintroduction risk. However, they should not be used uncritically as substitutes for parasite population structure in the presumed source settings. The present analyses further indicate that this caution applies not only to the epidemiological pathway through which imported cases are detected, but also to the construction and analysis of genomic datasets. Reference-panel composition, genotype filtering, missingness, within-host infection complexity, and the retained variant set can affect the resulting population-genomic summaries. Accordingly, imported and endemic-reference datasets should be compared using transparent, analysis-specific quality-control criteria and interpreted together with epidemiological information and the limitations of the available reference panel. This concern is not unique to malaria: pathogen genomic surveillance more generally is sensitive to how samples are selected and processed, which can influence variant detection, estimation accuracy, and the public-health information generated (Toyama et al., 2020).

Several limitations should be considered. First, the China-imported cohort was shaped by travel, symptom onset, healthcare seeking, diagnosis, reporting, and specimen availability. It therefore cannot represent all P. falciparum infections circulating in West Africa. In addition, the reported country of infection was based on epidemiological investigation and may not identify the precise location at which infection was acquired (Tatem et al., 2017). Second, although we used country-balanced subsampling, a geographically restricted West African reference panel, stricter genotype filtering, and F_WS_ restricted sensitivity analyses, these approaches did not fully harmonize the compared datasets. Differences in collection periods, local sampling schemes, sequencing characteristics, historical variant-calling procedures, and unmeasured population structure may still have contributed to the observed patterns. Accordingly, this study cannot quantify the independent contribution of the surveillance pathway relative to these factors. Third, only 9 and 4 China-imported isolates met the F_WS_>0.90 and F_WS_>0.95 thresholds, respectively. The D2b and D2a analyses therefore provided sensitivity assessments rather than replacement population samples. In particular, the D2a analysis was limited to qualitative assessment of broad population placement and was not designed for formal population-level inference or country-level source assignment. Finally, gene-level F_ST_ and Tajima’s D estimates were sensitive to the revised filtering and sample-restriction scheme. These statistics, especially in highly polymorphic and multi-copy gene families such as *var*, *rifin*, and *stevor*, may be affected by short-read mapping, callability, missingness, and differences in retained variants. We therefore did not use gene-level extremes as stand-alone evidence of selection, population-specific adaptation, or surveillance-specific biological change. Larger, temporally matched datasets generated and processed through comparable protocols, together with representative endemic-country sampling, will be needed to separate surveillance-related sampling effects from geographic and technical sources of variation.

## 5 Conclusion

Genomic data from imported malaria cases provide a valuable surveillance resource in malaria-free and post-elimination settings. They can support regional population placement, monitoring of parasite movement, updating of drug-resistance information, and assessment of reintroduction risk. However, imported-case genomes are generated through a movement- and detection-defined sampling process and should not be assumed to represent random or directly interchangeable samples of parasite populations circulating in presumed source settings.

In this study, China-imported West Africa-associated Plasmodium falciparum isolates remained distinguishable from the selected West African comparison datasets after country-balanced reference subsampling, geographically restricted comparison, stricter quality filtering, and restriction to high F_WS_ imported subsets. These analyses support the broad observation that imported surveillance genomes capture a particular representation of West African parasite diversity. At the same time, gene-level differentiation estimates and Tajima’s D summaries were sensitive to filtering, reference-panel composition, and within-host infection complexity.

Therefore, imported malaria genomes should be interpreted as surveillance-derived data rather than as direct proxies for endemic parasite populations. Cross-dataset comparisons should report sampling composition, collection period, sequencing and variant-filtering procedures, genome coverage, missingness, and infection complexity, and should evaluate the robustness of key findings under alternative reference panels and quality-control criteria. The observed differences in this study are compatible with differences in sampling and dataset-construction frameworks, but cannot be attributed solely to surveillance pathway. Integrating imported-case genomes with representative, geographically and temporally comparable endemic-country sampling will be necessary for robust inference of source-population structure.

## Data Availability

The datasets presented in this study can be found in online repositories. The names of the repository/repositories and accession number(s) can be found below: https://www.ncbi.nlm.nih.gov/, PRJNA1462630.

## Supplementary material

Supplementary Table 1 Epidemiological and genomic summary information of China-imported *P. falciparum* isolates.

Supplementary Table 2 Country-balanced reference-panel subsampling and principal component analysis results for the R2 sensitivity analysis. **(A)** Sample composition of the 20 country-balanced *Pf3K* reference-panel subsampling replicates used in the R2 sensitivity analysis. **(B)** Eigenvalues and variance explained by principal components across the 20 R2 subsampling replicates.

Supplementary Figure 1 Sensitivity of PCA placement of China-imported isolates to country-balanced random subsampling of the reference panel (R2 analysis). In the R2 sensitivity analysis, Pf3K reference isolates from 13 countries were randomly subsampled to a maximum of 30 isolates per country, and PCA was repeated for 20 independent sampling rounds. Panels show representative PCA results from rounds 1, 5, 10, and 20. Blue points indicate randomly selected Pf3K reference isolates; orange, grey, yellow, and dark-blue points indicate Sierra Leone local isolates, China-imported isolates, Togo local isolates, and previously reported Ghana-associated imported isolates, respectively. Across the 20 random subsampling rounds, China-imported isolates retained a broadly similar position relative to the African reference samples, although the orientation of principal components and the proportions of variance explained by individual components varied among rounds.
