# Supplementary material for: Interpreting imported malaria genomes: population-genomic sensitivity analyses of West Africa-associated Plasmodium falciparum isolates detected in China
Source: Front Cell Infect Microbiol. 2026 Oct 12;16:1950822. doi: 10.3389/fcimb.2026.1950822 (PMC13696243; doi:10.3389/fcimb.2026.1950822)
Supplement: Supplementary file 1 [file SupplementaryFile1.docx]

# Supplementary Note

Supplement to: Xuli Du, et al. **Interpreting imported malaria genomes: population-genomic sensitivity analyses of West Africa-associated Plasmodium falciparum isolates detected in China**

## **1 Overview of analytical datasets**

China-imported isolates and *Pf3K* reference samples were generated through different original sequencing and variant-calling workflows. We therefore used a tiered analysis framework to assess the sensitivity of population-genomic comparisons to reference-panel composition, genotype filtering, and within-host infection complexity. The original dataset was retained for comparison with the revised analyses. R2 assessed the effect of repeated country-balanced *Pf3K* subsampling on PCA. D1 was used for the principal population-structure analysis. D2a restricted the China-imported dataset to isolates with F_WS_>0.95 for a high-F_WS_ population-placement assessment. D2b restricted the China-imported dataset to isolates with F_WS_>0.90 for diversity, differentiation, and allele-frequency-spectrum analyses. Dataset composition, filtering, and intended analyses are summarized in Supplementary Methods Table.

| ***Dataset*** | ***Sample composition*** | ***Variant set and filtering*** | ***Purpose and analyses*** |
| --- | --- | --- | --- |
| Original | 31 China-imported isolates and original reference panel | Original filtering: DP≥3; QC-pass; no core-position restriction; | Initial population-structure, π\piπ, gene-level F_ST_ , and Tajima’s D comparisons |
| R2 | Fixed comparison set: 31 China-imported + 4 Sierra Leone local + 7 Togo local + 9 Ghana-associated imported; plus Pf3K samples from 13 countries, ≤30/country, 20 random replicates | Same filtering framework as original; [shared SNP 73,500 / total loci 558,707] | Sensitivity of PCA to Pf3K reference-panel subsampling |
| D1 | 31 China-imported + 150 Pf3K from five West African countries + 4 Sierra Leone local + 7 Togo local + 9 Ghana-associated imported; total n=201 | DP≥5; QC-pass; MAF≥0.05; PLINK LD-pruned; no core-position restriction; final shared SNP n=53,710 | PCA, maximum-likelihood phylogeny, and STRUCTURE |
| D2a | 4 China-imported, F_WS_>0.95 + 150 Pf3K from the same five West African countries; total n=154 | DP≥5; QC-pass; core-position filter; MAF≥0.05; PLINK LD-pruned; final shared SNP n=30,351 | Qualitative high-F_WS_ PCA and phylogenetic sensitivity analysis |
| D2b | 9 China-imported, F_WS_>0.90>0.90 + 150 Pf3K from the same five West African countries; total n=159 | DP≥5; QC-pass; core-position filter; no MAF filter; no LD pruning; final shared SNP n=84,590 | π, gene-level F_ST_ , and Tajima’s D sensitivity analyses |

“Total loci” denotes all retained loci in the integrated VCF after the stated filter. “Shared SNPs” denotes biallelic SNPs retained in both the China-imported and *Pf3K* datasets after coordinate and allele harmonization. Population-structure and population-genetic analyses used the shared SNP set specified for each dataset.

## **2 Variant harmonization and quality filtering**

China-imported isolates and *Pf3K* reference samples were not assumed to have been generated through identical historical variant-calling pipelines. Independent, dataset-specific stringent filtering substantially reduced the number of SNPs shared between the two callsets, whereas union-based variant sets retained many sites available in only one dataset. Such sites were not used for joint population-structure analyses because dataset-specific variant availability may influence leading axes of PCA or phylogenetic reconstruction.

We therefore harmonized the datasets using shared biallelic SNPs and the quality annotations available in the *Pf3K* release VCF. Indels and multiallelic sites were excluded. Variants failing the applicable VQSR/VQSLOD or variant-quality filters were removed. For the revised analyses, China-imported samples were required to have a parasite-mapping proportion of at least 90%. Samples and sites with missing genotype proportions greater than 0.02 were excluded. The original analysis used DP ≥ 3, whereas D1, D2a, and D2b used DP ≥ 5. Further restrictions, including MAF filtering, core-position filtering, and LD pruning, were applied according to the purpose of each analysis as described below.

## **3 Original analysis and R2 subsampling**

**Original analysis**

The original analysis included 31 China-imported isolates and the corresponding original reference dataset. Genotypes were filtered using DP ≥ 3 and the original QC framework. This dataset was used for the initial comparisons of population structure, nucleotide diversity, gene-level F_ST_, and Tajima’s D. It was retained as the baseline for comparison with the R2, D1, D2a, and D2b sensitivity analyses.

**R2: country-balanced reference-panel subsampling**

R2 evaluated whether the broad PCA placement of China-imported isolates was dependent on the selection of Pf3K reference samples. In each of 20 independent replicates, a maximum of 30 *Pf3K* isolates was randomly selected from each of 13 countries. The fixed comparison set consisted of 31 China-imported isolates, four locally collected Sierra Leone isolates, seven locally collected Togo isolates, and nine previously reported Ghana-associated imported isolates. R2 used the same filtering framework as the original analysis. The integrated dataset contained 558,707 loci before harmonization, of which 73,500 were shared SNPs. PCA was performed for each replicate. The sample composition of all 20 replicates is listed in Supplementary Table S2.

## **4 D1 population-structure analysis**

D1 included 31 China-imported isolates; 150 *Pf3K* isolates randomly selected from Gambia, Guinea, Ghana, Mali, and Senegal (30 per country); four locally collected Sierra Leone isolates; seven locally collected Togo isolates; and nine previously reported Ghana-associated imported isolates. The D1 dataset therefore contained 201 isolates. Genotypes were filtered at DP ≥ 5 under the shared QC framework. After initial harmonization, the dataset contained 408,392 loci, including 69,226 shared SNPs. Following MAF ≥ 0.05 filtering, 272,300 loci remained, including 53,710 shared SNPs used for downstream analysis. No additional core-position filter was applied in D1, to permit direct comparison with the original structure analysis. The final shared SNP set was LD pruned using PLINK2 before PCA, maximum-likelihood phylogenetic reconstruction, and STRUCTURE analysis.

## **5 D2a high-F_WS_ population-placement analysis**

D2a included four China-imported isolates with F_WS_>0.95 and 150 *Pf3K* isolates selected from Gambia, Guinea, Ghana, Mali, and Senegal (30 per country), for a total of 154 isolates. Togo, Sierra Leone, and the previously reported Ghana-associated imported comparison isolates were not included in D2a. Genotypes were filtered at DP ≥ 5 under the shared QC framework and were restricted using the core-position filter. The initial integrated dataset contained 252,280 loci, including 62,226 shared SNPs. After MAF ≥ 0.05 filtering, 55,666 loci remained, including 30,351 shared SNPs. The final shared SNP set was LD pruned using the same PLINK framework as D1 and used for qualitative PCA and phylogenetic assessment of the high-F_WS_ China-imported subset.

## **6 D2b diversity, differentiation, and Tajima’s D analysis**

D2b included nine China-imported isolates with F_WS_>0.90 and 150 *Pf3K* isolates selected from Gambia, Guinea, Ghana, Mali, and Senegal (30 per country), for a total of 159 isolates. Genotypes were filtered at DP ≥ 5 under the shared QC framework and were restricted using the core-position filter. The integrated dataset contained 332,080 loci, including 84,590 shared SNPs. No MAF filter or LD pruning was applied, because nucleotide diversity, gene-level F_ST_ , and Tajima’s D depend on the observed distribution of segregating sites and allele frequencies. D2b was used to compare π\piπ, gene-level F_ST_ , and Tajima’s D with the corresponding results from the original analysis.

## **7 Supplementary files and reproducibility resources**

The supplementary files provide the scripts, workflow record, and *Pf3K* reference documentation used to support dataset construction and quality control. These materials are provided to clarify the analytical procedures and the provenance of the *Pf3K*-derived annotations and filters. They do not include complete raw sequencing data or the complete *Pf3K* release files, which are available from their original sources as described in the *Pf3K* documentation.

**Author-generated scripts and workflow record.** extract_pf3k_columns.pl was used to select *Pf3K* samples for the country-balanced random subsampling procedure used in the R2 sensitivity analysis. filter_VQSLOD.pl was used to retain variants according to the specified VQSLOD threshold during quality filtering. pipeline-west African imported samples.txt provides the command-level workflow record for processing the China-imported samples, including read alignment, duplicate marking, local realignment, base-quality score recalibration, variant calling, variant quality score recalibration, functional annotation, and subsequent variant filtering.

***Pf3K* documentation and reference resources.** pf3k_5_1.README.txt is the accompanying documentation for the Pf3K release used in this study. It describes the release files, variant annotations, and VQSLOD-based filtering framework, and provides information on access to the associated recalibration resources. regions-20130225.onebased.txt is the *Pf3K* genomic-region annotation file used during snpEff annotation to assign region classes, including core, centromeric, internal hypervariable, subtelomeric hypervariable, and subtelomeric repeat regions. In the *Pf3K* VCF, the corresponding RegionType annotation defines “Core” as genomic sequence outside the centromeric, internal hypervariable, subtelomeric hypervariable, and subtelomeric repeat categories.

**Gene annotation summary.** snpEff_genes.txt is a tab-delimited, gene-level summary of snpEff functional annotations for the *P. falciparum* 3D7 reference genome. For each gene and transcript, it records the gene biotype and the numbers of annotated variants in each impact category and effect class. This file was used as an annotation reference for downstream gene-level summaries and interpretation.

**Illustrative Pf3K VCF excerpt.** SNP_INDEL_Pf3D7_01_v3.combined.filtered.vcf100.txt contains the first 100 lines of the chromosome 1 filtered VCF. It is supplied as an illustrative excerpt of the VCF header, metadata, INFO annotations, sample fields, and provenance information; it is not the complete VCF used for analysis. The header documents the release’s GATK VariantFiltration process, including filtering of sites with VQSLOD < 0 and annotations of non-core region types. It also defines VQSLOD as the log odds that a variant is true rather than false under the trained Gaussian mixture model.

| File | Type | Role in this study |
| --- | --- | --- |
| extract_pf3k_columns.pl | Perl script | Country-balanced random selection of Pf3K samples for the 20 R2 replicates |
| filter_VQSLOD.pl | Perl script | VQSLOD-based retention of variants during QC |
| pipeline-west African imported samples.txt | Workflow record | Command-level record of processing and filtering of China-imported sequencing data |
| pf3k_5_1.README.txt | Pf3K documentation | Release documentation, variant annotations, VQSLOD framework, and instructions for related recalibration resources |
| regions-20130225.onebased.txt | Pf3K region annotation | Genomic-region annotations used during snpEff annotation and definition of core/non-core genomic regions |
| snpEff_genes.txt | Gene annotation summary | Gene- and transcript-level counts of snpEff impact and effect annotations |
| SNP_INDEL_Pf3D7_01_v3 combined.filtered.vcf100.txt | Illustrative VCF excerpt | First 100 lines of the Pf3K chromosome 1 filtered VCF, provided to document header metadata and VCF annotations |
